# Association between serum total testosterone and Body Mass Index in middle aged healthy men

**DOI:** 10.12669/pjms.312.6130

**Published:** 2015

**Authors:** Muhammad Omar Shamim, Farooq Munfaet Ali Khan, Rabia Arshad

**Affiliations:** 1 Dr. Muhammad Omar Shamim, MBBS, MPhil. Assistant Professor, Physiology Department, Islam Medical College, Pasrur Road, Sialkot, Pakistan; 2 Dr. Farooq Munfaet Ali Khan, MBBS, MPhil. Assistant Professor, Physiology Department, Mohtarma Benazir Bhutto Shaheed Medical College, MirPur, Azad Jammu Kashmir, Pakistan; 3 Dr. Rabia Arshad, MBBS, M. Phil. Assistant Professor, Pharmacology Department, Sir Syed College of Medical Sciences, Clifton Karachi, Pakistan

**Keywords:** Serum Total Testosterone, BMI, Waist Hip Ratio

## Abstract

**Objective:**

To determine correlation of serum total testosterone with body mass index (BMI) and waist hip ratio (WHR) in healthy adult males.

**Methods:**

A cross sectional study was conducted on 200 nonsmoker healthy males (aged 30-50 years) university employees. They were selected by convenience sampling technique after a detailed medical history and clinical examination including BMI and Waist Hip Ratio (WHR) calculation. Blood sampling was carried out to measure serum total testosterone (TT) using facilities of Chemiluminescence assay (CLIA) technique in Dow Chemical Laboratory. Independent sample T test was used for mean comparisons of BMI and WHR in between low and normal testosterone groups. (Subjects having < 9.7 nmol/L of total testosterone in blood were placed in low testosterone group and subjects having ≥ 9.7 nmol/L of total testosterone in blood were placed in normal testosterone group). Correlation of testosterone with BMI and WHR was analyzed by Pearson Correlation.

**Results:**

Mean (± SD) age of the subjects included in this study was 38.7 (± 6.563) years mean (± SD) total testosterone was 15.92 (±6.322)nmol/L. The mean (± SD) BMI, and WHR were 24.95 (±3.828) kg/m^2^ and 0.946 (±0.0474) respectively. Statistically significant differences were observed in the mean values of BMI and WHR for the two groups of testosterone. Significant inverse correlation of serum total testosterone with BMI(r = -0.311, p = 0.000) was recorded in this study. However testosterone was not significantly correlated with waist/hip ratio.(r = -0.126, p = 0.076)

**Conclusion:**

Middle age men working at DUHS who have low level of serum total testosterone are more obese than individuals with normal total testosterone level.

## INTRODUCTION

With the advancement of life in the developing countries there is a visible change noticed in the life style including lack of physical activity along with increased junk food intake. Over the last two to three decades, this change resulted in increased risk of overweight and obesity.^1^

Low testosterone levels along with the proven effects on male secondary sexual characteristics also increase body mass index^2^ which is primarily due to overweight and obesity. Obesity is the root cause of many systemic illnesses like diabetes mellitus and cardiovascular diseases and directly associated with low Total Testosterone levels.^3^

The probable mechanism by which obesity results due to low testosterone is the alteration of protein and fat metabolism by testosterone. Under physiological conditions, testosterone is associated with significant rise in muscle mass providing maximal voluntary strength and decreased fat mass.^4^ It decreases fat mass by mechanisms acting at different steps in fat metabolism such as inhibiting the activity of lipoprotein lipase,^5^ and inhibiting the activity of glyceraldehyde 3-phosphate dehydrogenase.^6^ Besides this, dihydrotestosterone specifically causes decreased lipid accumulation and enhanced lipolysis in fat cell precursors.^7^ Moreover, testosterone and DHT modulate the mesenchymal stem cell differentiation in a way that adipocytes differentiation is inhibited and shifted towards the formation of myogenic cells.^8^

Although low total testosterone levels have been associated with increased body mass index which is mainly attributed to obesity as it is evident from the above paragraph. Studies have also suggested that low total testosterone levels may be related to metabolic syndrome.^3^ However the association of total testosterone and body mass index in healthy subjects aged between 30 to 50 years has been examined rarely, as most of the studies were carried out in older age (> 50 years) group and on diseased subjects. Therefore we examined the association of total testosterone with body mass index in healthy adult male employees of Dow University of Health Sciences at Karachi.

## METHODS

The study design was cross sectional and was carried out in three medical colleges of Dow University in collaboration with Dow Chemical Lab from August 2010 to May 2011. We recruited 200 healthy nonsmoker male University employees aged between 30-50 year selected by convenience sampling technique from the university male employees list. The age group between 30 – 50 years is considered to be middle age as it was the same age group in Massachusetts Male Aging Study in which prevalence and incidence of androgen deficiency in Middle-aged and older men were estimated.^9^

Sample size was calculated with the guidance of bio-statistician and epidemiologist through software (open epi). For which the prevalence of androgen deficiency was taken as 12.3%, estimated from Massachusetts Male Aging Study.^9^ We did not include smokers as variations in testosterone levels were have been reported in smokers.^10^ In addition subjects suffering from any acute, chronic and systemic illnesses, taking any testosterone supplementation, suffering from hypogonadism and those who indulged in regular exercise^11^ were also not included in the study.

A total of 245 individuals were interviewed during 2010 - 2011. Out of these 245 individuals, 26 were smokers, 17 were suffering from systemic diseases (11 had Diabetes along with hypertension, 4 were suffering from ischemic heart disease, 2 had bronchial asthma) and were excluded on the basis of detailed history and clinical examination. In addition, 2 participants gave consent but did not come for sampling. So they were also excluded from the study. This eventually led to the final sample size of 200.

Informed written consent was taken from the subjects on a prescribed consent form approved by Dow University Institutional Review Board, followed by a detailed medical history and thorough clinical examination recorded on a prescribed proforma approved by Institutional Review Board. Anthropometric measurements (height, weight, BMI, waist and hip circumference and waist hip ratio) were calculated using standard techniques. Calculation of BMI was done by the formula (BMI = weight in Kilogram / Height in meter square). BMI was categorized according to the new Asian classification of BMI.^12^ Waist margin was determined at a point midway between the margin of the lower rib and iliac crest with the help of measuring strip surrounding the body horizontally. Hip circumference was measured at the level of the greatest protrusion of the gluteal (buttock) muscles. Blood samples were collected by standardized venipuncture technique in the morning as testosterone shows diurnal variation.^13^ Later it was tested in Cobas e 411analyzer manufactured by Hitachi in Dow lab by Chemiluminescence assay (CLIA) technique, followed by competitive test principle,^14^ in which 20 micro liter of sample was incubated with biotinylated monoclonal testosterone specific antibodies resulting in competitive binding of an unlabeled antigen present in sample and an enzyme labeled antigen (conjugate) for a limited number of antibody binding sites on the microwell plate. Streptavidin coated micro particles and a testosterone derivative labeled with a ruthenium complex was added. Reaction mixture was aspirated into the measuring cell where micro particles were magnetically captured onto the surface of electrodes. Voltage was applied inducing chemiluminescent emission and was measured by photomultiplier. Range of detection was 0.087 - 52.0 nanomole per liter (nmol/L).

The total duration of assay was of 18 minutes. Cut off value for Serum Total Testosterone (Adult Male) was 9.7nmol/L, which was according to the international standard used by Dow chemical lab.^15^ Data were analyzed using SPSS version 16. Serum total testosterone reference values were categorized into two groups on the basis of lab results < 9.7 nmol/L (Group 1 having low total testosterone levels) and ≥ 9.7 nmol/L (Group 2 having normal total testosterone levels). Descriptive statistics including mean, standard deviation, range and frequency were computed for all the variables including age, testosterone, BMI, Waist-Hip Ratio (WHR). Independent sample T test was used to compare the means of all variables for significance of difference. Pearson correlation was applied to determine co-relation and its strength between testosterone and BMI along with WHR. Threshold for statistical significance was set at p < 0.05.

## RESULTS

Table-I shows descriptive statistics of 200 healthy subjects aged between 30 to 50 years. Mean (±SD) BMI was 24.95 (±3.828) kg/m^2^ indicative of overweight range of BMI classification for Asian population.

**Table I:** Descriptive Statistics of 200 healthy male subjects.

Variables	Mean	Standard Deviation	Minimum	Maximum	Range
Age (Years)	38.72	6.563	30	50	20
BMI (kg/m2)	24.95	3.828	14.23	35.87	21.64
Waist (inch)	35.97	3.365	26	48	22
Hip (inch)	38.01	2.996	30	52.4	22.4
Waist-Hip Ratio	0.946	0.0474	0.81	1.17	0.36
Testosterone nmol/L)	15.9	6.322	5.63	43.64	38.01

For mean comparison of BMI and WHR testosterone values were categorized into two groups (Low and normal) on the basis of Dow chemical lab reference value, in such a way that out of 200 healthy study subjects 27 were having low testosterone levels and 173 had normal levels.

Mean comparison in between two groups (Low and normal) of total testosterone for body mass index and waist hip ratio was analyzed by applying Independent Samples T Test. Statistically significant (p < 0.05) differences were observed in the mean values of BMI and WHR for the two groups of testosterone as shown in Table-II.

**Table-II:** Mean Comparison by Independent Samples T Test.

Test Variables	Low Testosterone Group	Normal Testo Group	P Value
Mean±SD BMI	26.48 ± 3.635	24.71 ± 3.812	*0.025
Mean±SD WHR	0.97 ± 0.032	0.94 ± 0.048	*0.004

BMI comparison (according to the new Asian BMI classification by WHO)^12^ between low and normal groups of total testosterone in present study is shown in Table-III.

**Table-III:** BMI Comparison between Low and Normal Testosterone Groups.

New Asian BMI Classification(kg/m^2^)	Low Testosterone Group (n = 27)	Normal Testosterone Group (n = 173)
Underweight (< 18.5)	Nil	9 (5.2%)
Normal BMI(18.5 - 22.9)	5 (18.5%)	49 (28.3%)
Overweight(23 – 24.9)	7 (25.9%)	29 (16.7%)
Obese(≥ 25)	15 (55.5%)	86 (49.7%)

To measure the strength of the linear relationship between testosterone and BMI Pearson Correlation was applied as shown in Table-IV.

**Table-IV:** Correlation of Testosterone with BMI and Waist-Hip Ratio (n=200).

	Correlation Coefficient	P Value
BMI	-0.311	*0.000
Waist Margin	-0.311	*0.000
Hip Margin	-0.295	*0.000
Waist-Hip Ratio	-0.126	0.076

Pearson Correlation was applied,

* Significant(p < 0.05)

## DISCUSSION

Androgen deficiency in males (mainly total testosterone) has been investigated in several parts of the world in recent years. The frequency of low serum total testosterone in Dow University male employees aged 30 to 50 years, recorded in this study was 13.5%. Previously Goel *et al.*, found 24.2% frequency of low total testosterone in Indian healthy population aged 40 to 60 years.^16^ The mean total testosterone in this study was 15.92 nmol/L which is consistent with mean testosterone of 14.6 nmol/L reported by Heald *et al.*, for Pakistani men residing in England.^17^

Obesity is broadly documented as an essential public health problem; its prevalence has increased significantly in the recent decades. BMI and waist hip ratio measurements are one of the important tools to assess obesity and mostly used in studies evaluating the relationship between total testosterone and obesity.

Overall 68.5% of the study subjects (n = 200) in the present study were overweight and obese (had BMI more than 22.9 kg/m^2^) while 50.5% of all the study population were obese (had BMI more than 24.9 kg/m^2^), similarly Jafar *et al.*, reported the prevalence of overweight and obesity in 4414 Pakistani men above 15 years from National Health Survey (1990-1994) as 22%, giving 12.5% prevalence of obesity.^18^

Another health survey in England^19^ showed 15% Pakistani men above 16 years of age were obese type II (had a BMI of 30 kg/m^2^ and over), compared to the present study which reported 9.5% of all the subjects (n = 200) as obese type II. It also reported 36% of Pakistani men had WHR of 0.95 or more and 30% had waist circumference of 40.2 inches or more,^19^ while the present study showed 41% of all the study population (n = 200) had WHR of 0.95 or more and 7% of all the study subjects (n = 200) had waist circumference of 40.2 inches or more and were at high risk according to American college of sports medicine (ACSM) guidelines.^20^

Overall mean waist circumference and WHR in the present study was 36 (±3.365) inches and 0.94(± 0.0474) respectively, which is slightly different with mean waist circumference and WHR of 37.4 inches and 0.92 respectively reported in England health survey for Pakistani men.^19^

Mean comparison of BMI and WHR in between the two groups of testosterone in present study showed significant mean difference in both the testosterone groups (p = 0.025 and p = 0.004, respectively).

Another study also showed significant mean differences of BMI (p <0.01) and WHR (p < 0.001) in men (aged 20-60 years) categorized in low and normal Testosterone groups.^21^ These findings were similar to the current study results.

Furthermore in a longitudinal cohort study Gapstur *et al.*, determined changes in total testosterone, free testosterone and sex hormone binding globulin(SHBG) levels linked with changes in body mass index during young, adulthood and modulate age.^22^ Osuna and his colleagues evaluated the relationship of BMI and TT in 77 men aged between 20 to 60 years in a cross sectional study, which were categorized in three BMI groups (normal, overweight and obese).^23^ They found significant negative correlation of TT with BMI in the obese group then the normal and overweight group,^23^ suggesting a mechanistic link between and TT and BMI. Osuna’s findings were similar to the present study results which also showed significant negative correlation of TT with BMI. Another survey based study (in 1548 men in between 25–84 years of age), showed significant (p< 0.001) inverses correlation between TT and waist circumference, while insignificant correlation was recorded between TT and WHR.^24^ These findings were quite similar to the current study results which also showed significant (p = 0.000) inverse correlation of TT with waist circumference and insignificant correlation of TT with WHR (p = 0.076).

Therefore we believe that these inverse correlations between TT, BMI and waist circumference are responsible for the modulation of the lean body mass, fat mass and body composition due to low TT.

### Limitations of the study

It was conducted at one institution with a comparatively small sample size and measurements were also done only once.

## CONCLUSION

Serum total testosterone has a significant negative correlation with BMI of middle aged men working at Dow University.
